# Supplementary material for: Soil Fungal Community Differences in Manual Plantation Larch Forest and Natural Larch Forest in Northeast China
Source: Microorganisms. 2024 Jun 28;12(7):1322. doi: 10.3390/microorganisms12071322 (PMC11278968; doi:10.3390/microorganisms12071322)
Supplement: Supplementary file 1 [file microorganisms-12-01322-s001.zip › microorganisms-3050615-supplementary.pdf]

Table S1. Soil physicochemical properties in different treatments (NR, MR).

Sample	Mean(NR)	Variance(NR)	Stderr(NR)	Mean(MR)	Variance(MR)	Stderr(MR)	P	Fdr
AK	68.17166667	2.830456667	0.686835335	43.18666667	4.556986667	0.871491697	6.71644E-10	2.2731E-09
AN	95.61833333	14.93921667	1.577932015	168.24	50.5776	2.903377344	8.52411E-10	2.2731E-09
TK	11.55666667	1.463891367	0.493945234	4.607133333	0.413604299	0.262552947	2.10704E-07	4.21409E-07
PH	5.838333333	0.260496667	0.208365331	3.313333333	0.188426667	0.177212992	3.29283E-06	5.26852E-06
TP	0.507833333	0.012507995	0.04565814	0.173183333	0.00157425	0.016197992	4.15549E-05	5.54065E-05
AP	6.464133333	0.396869519	0.257186547	4.313833333	0.381736875	0.252235629	0.000137658	0.000157323
TN	2.428333333	0.150829867	0.158550658	1.585666667	0.051432267	0.092585336	0.000995872	0.000995872

Note: MR: Manual recovery larch forest, NR: Natural larch forest. Six replications were performed for each treatment. The data shows the mean, variance and stderr of different treatment groups ($P<0.05$). The table displays the P-values and fdr. AK: Available Potassium. AN: Available nitrogen. TN: Total nitrogen. TP: Total phosphorus. TK: Total potassium. AP: Available phosphorus. SOC: soil organic carbon. pH: Pondus hydrogenii.

Table S2. Alpha diversity of fungal communities under different treatments (NR, MR).

Sample	NR	MR	p-Value	fdr
Observed_species	250.1667±94.311	313.3333±25.2877	0.003455	0.005781667
Chao1	250.1771±94.3268	313.3611±25.2662	0.00345	0.005781667
ACE	250.3356±94.3693	313.4617±25.1947	0.003469	0.005781667
Shannon	3.5439±0.5148	4.0981±0.3126	0.038155	0.038155
Simpson	0.9089±0.0526	0.9566±0.0181	0.010977	0.01372125

Note: MR: Manual recovery larch forest, NR: Natural larch forest Six replications were performed for each treatment. The data are expressed as the mean ± standard deviation. The table displays the P-values and fdr.

Table S3. Topological parameters of fungal communities networks in different treatments (NR, MR).

Sample	Node number	Edge number	Average degree	Eigenvector Centrality	Modularity index	Networking Density	statistical inference
NR	215	1177	10.949	0.04189	0.832	0.051	4262.863
MR	243	239	6.457	0.02354	0.862	0.033	2416.658

Note: Node is the basic elements in a network diagram, and in microbial network analysis, a node represents a species or OUT. Node number indicates the number of nodes in the network. Edges are the connecting lines between nodes and represent the relationship or connection between nodes. Edges number indicates the number of edges in the entire network. The average degree is the sum of the degrees of all nodes in the network divided by the number of nodes is the average degree, which reflects the average interaction strength of the microorganisms in the network. Eigenvector centrality is a class of metrics used to measure the importance and influence of nodes in a network. Modularity index refers to the number and structure of modules made up of nodes in a network. Networks with high modularity have a distinct substructure, whereas networks with low modularity have less of such a structure. Network Density is the ratio of the number of edges actually present in the network to the maximum number of edges possible. Network density can reflect the tightness and complexity of the network. MR: Manual recovery larch forest, NR: Natural larch forest
